# Body composition measurements

**DOI:** 10.1186/1824-7288-40-S1-A6

**Published:** 2014-08-11

**Authors:** Angelo Pietrobelli, Paolo Brambilla

**Affiliations:** 1 Pediatric Unit, Verona University Medical School, Verona, Italy; 2 Pennington Biomedical Research Center, Baton Rouge, LA, USA; 3 Azienda Sanitaria Locale (ASL) Milano 2, Melegnano, Milan, Italy

## 

Accurate assessment of body composition is important in obesity and in many areas of nutrition-related research. The foundation of the development of body composition analysis has been the two-compartment model, dividing body mass into fat mass (FM) and fat-free mass (FFM). A range of techniques are available. Some of them are producing very accurate data, so called “gold standard” or reference methods, have disadvantages of cost, limited availability. Simpler techniques are well tolerated, portable and therefore can be employed in the clinic, patient’s bedside or in the community, though may be less accurate. Densitometry determines body density by measuring weight after whole-body immersion in water. It has been crucial as a reference technique in development of body composition studies. Computed Tomography (CT) and Magnetic Resonance Imaging (MRI) provide anatomical detail and can be used to assess skeletal muscle volume and measure the intra-abdominal fat depot. Dual energy X-ray Absorptiometry (DXA) measures the relative attenuation of two different energy X rays by the body. It derives a three-compartment model of body composition, FM, lean and bone mineral. Lean tissue measured by DXA includes body water, and changes in hydration will be reflected by DXA as change in lean tissue. Anthropometry is a simple technique. Skinfold thickness measurement allows estimation of body fat content. Body density and, thus, subcutaneous body fat can be estimated from the sum of skinfold thicknesses. Waist circumference-to-height ratio is now the most preferred anthropometric index due to its ability to estimate both total adiposity and fat distribution. Weight-stature indices provide surrogate measures for body composition measurements. The most widely used weight-stature index is Body Mass Index (BMI, weight/height^2^, kg/m^2^). BMI is a global index of nutritional status, but its relation with body composition *per se* is controversial. BMI is a poor predictor for individual subject in clinical setting, because is not able to disentangle FM *Vs* FFM. Bioimpedance analysis (BIA) is a potential field and clinical method for evaluating %fat and skeletal muscle mass (SM). It offers the advantages of portability, compactness, economy, and ease of operation. Total body water in turn can be used to derive fat and fat free mass. In conclusion, among several possible applications, we want to underline the validity of waist circumference and BIA in the day by day clinical practice as clear, simple and useful measurements of pediatric body composition.

